# PU.1 upregulation is linked to improved prognosis in non-small cell lung cancer

**DOI:** 10.3389/fimmu.2025.1604237

**Published:** 2025-08-15

**Authors:** Katja Hohenberger, Denis I. Trufa, Arndt Hartmann, Susanne Mittler, Sonja Trump, Horia Sirbu, Susetta Finotto

**Affiliations:** ^1^ Department of Molecular Pneumology, Friedrich-Alexander-Universität (FAU) Erlangen-Nürnberg, Universitätsklinikum Erlangen, Erlangen, Germany; ^2^ Department of Thoracic Surgery, Friedrich-Alexander-Universität (FAU) Erlangen-Nürnberg, Universitätsklinikum Erlangen, Erlangen, Germany; ^3^ Institute of Pathology, Friedrich-Alexander-Universität (FAU) Erlangen-Nürnberg, Universitätsklinikum Erlangen, Erlangen, Germany; ^4^ Bavarian Cancer Research Center (BZKF), Erlangen, Germany; ^5^ Comprehensive Cancer Center Erlangen-EMN (CCC ER-EMN), Erlangen, Germany; ^6^ Deutsches Zentrum für Immuntherapie (DZI), Erlangen, Germany

**Keywords:** non-small cell lung cancer, NK cells, PU.1, recurrence free survival (RFS), metastasis free survival (MFS)

## Abstract

**Background:**

Lung cancer is one of the most common types of cancer, which can currently be cured only partially. Even though the function of the transcription factor PU.1 as an oncogene has already been investigated in detail in various studies, its precise function and regulatory mechanisms in non-small cell lung cancer (NSCLC) remain to be fully elucidated.

**Methods:**

Patients with NSCLC and healthy controls were recruited at the Department of Thoracic Surgery of the University Hospital Erlangen. Tissue samples were dissected from patients who underwent lung tumor surgery and blood samples were collected from all participants.

**Results:**

In this study, we showed that the expression of PU.1 was increased both in the blood and in the tumor region of the lung of patients with NSCLC. On a more detailed analysis, PU.1 was found increased the most in CD56^dim^ natural killer cells (NK cells). Furthermore, we demonstrated an increased cytotoxic potential for PU.1 expressing cells as well as improved overall and recurrence-free survival for patients with a higher expression of PU.1, suggesting a beneficial role for PU.1 in NSCLC.

**Conclusion:**

High expression of PU.1 associated with NK cells could lead to a beneficial outcome and survival of NSCLC patients.

## Introduction

Lung cancer is the most common cancer type and the leading cause of cancer-related deaths worldwide (1). The different types of lung cancer can be histologically divided into non-small cell lung cancer (NSCLC) and small cell lung cancer (SCLC), with NSCLC being the major subtype accounting for 85% of lung cancer cases (2, 3). Several risk factors have been associated with the development of lung cancer. While tobacco smoking is the most significant cause (4–6), passive smoking and carcinogenic chemicals can increase the risk of lung cancer (7, 8). However, approximately 15-20% of lung cancer patients are never smokers or have smoked very little. In these patients, the cause of the disease is not always known; however certain genetic mutations or familial predispositions are suspected (9).

Depending on the tumor stage, the main treatment for NSCLC is surgical removal of the tumor, possibly followed by adjuvant therapy such as chemotherapy, chemoradiotherapy or immunotherapy (10, 11). Despite continuous improvements in the development of therapeutic options, treatment is not sustainably successful in all patients (12).

The immune system itself has the potential to destroy cancer cells and inhibit tumor growth by activating innate and adaptive immune responses. Innate immune responses develop quickly and are mediated by effector cells, such as natural killer cells (NK cells) and antigen-presenting cells like macrophages or dendritic cells (13). This leads to the secretion of perforin and granzyme, interferon gamma (IFN-γ) and other inflammatory cytokines to induce apoptosis of tumor cells (14, 15). An important transcription factor for the development of most immune cells is PU.1 (*SPI1*). Particularly the myeloid cell line is dependent on a high expression of PU.1, so that macrophages can develop from precursor cells and perform their function during an immune response. In addition, PU.1 is involved in the differentiation and proliferation of T cells, B cells and NK cells (16, 17). Point mutations or deletions in PU.1 have been associated with several diseases, such as the development of acute myeloid leukemia (AML) (18). As a result, upregulation of PU.1 is being considered as a therapeutic target in AML or Hodgkin’s lymphoma (19). In contrast, PU.1+ T helper 9 (Th9) cells have been found to accumulate in solid tumors, including experimental colorectal cancer (20) or lung cancer (21). Here, the deletion of IL-9 in T cells or the deactivation of PU.1 resulted in impaired tumor growth, suggesting a pro-tumoral role for PU.1+ Th9 cells (20, 21). While previous studies have focused primarily on the role of PU.1 in myeloid and T cell lineages, its involvement in the regulation of NK cells in the tumor microenvironment has received comparatively little attention. NK cells are critical effectors of innate anti-tumor immunity, capable of killing tumor cells without prior sensitization (22). Given the relevance of NK cells in tumor immune surveillance, and the potential for PU.1 to influence their anti-tumor activity, this study aims to investigate the role of PU.1 in NK cells in the context of NSCLC.

For this reason, we gained a deeper insight on the role of PU.1 in the blood and lung tumor tissue of patients with NSCLC and demonstrated that it plays an important role in the cytotoxic function of NK cells.

## Materials and methods

### Study design

This human study was conducted in collaboration with the Department of Thoracic Surgery of the University Hospital Erlangen. For blood donation, patients diagnosed with NSCLC were recruited by the clinical department of Thoracic Surgery and healthy control subjects were recruited by the department of Molecular Pneumology. Furthermore, patients with NSCLC underwent surgery and tissue samples were collected from the tumoral area (TU, solid tumor tissue), peritumoral area (PT, 2cm around the tumor), and a tumor-free control area (CTR, at least 5cm away from the solid tumor) of the surgically removed lung material. The patients included in this study were not treated with immunotherapy before undergoing surgery. Lung tissue samples were cut into three pieces and individually processed. One piece was directly used for cell isolation, one piece was stored in RNAprotect Tissue Reagent (QIAGEN) at -80°C and one piece was stored for protein isolation (-80°C). Histological subtype classification and TNM staging were confirmed by the Institute of Pathology of the University Hospital Erlangen. TNM staging was performed according to the eight (8^th^) edition of the TNM Classification for Lung Cancer in 2017. Relevant data were provided by the Department of Thoracic Surgery and the Institute of Pathology at the University Hospital in Erlangen and are shown in **Supplementary Table S1**.

### Study approval

This study was approved by the Ethics Review Board of the University Erlangen (Re-No: 56_12B and 22-64B; DRKS-ID: DRKS00029641 and DRKS00005376) and performed in accordance with the Declaration of Helsinki. Written informed consent was obtained from all patients and controls. Patient´s confidentiality was also maintained.

### RNA isolation

Total RNA was extracted from frozen tissue samples or peripheral blood mononuclear cells (PBMCs) using Qiazol Lysis^®^ Reagent (QIAGEN, Hilden, Germany), according to the manufacturer´s protocol. A small piece of lung tissue was added to 500 µl of Qiazol and homogenized. The homogenate (or in Qiazol stored PBMCs) was incubated at room temperature (RT) for 5 min. After that, 100 µl Choroform was added, the samples were vortexed for 20 seconds, incubated for 3 min (RT) and centrifuged for 15 min (12000 x g, 4°C). After centrifugation, the upper phase was transferred to a new tube and RNA was precipitated by the addition of 3 µl glycogen and 350 µl isopropanol. The samples were vortexed for 15 s, incubated for 15 min (RT) and centrifuged (10 min, 12000 x g, 4°C). The supernatant was discarded, and the pellet was washed with 1 ml of 70% ethanol (5 min, 12000 x g, 4°C). The remaining ethanol was discarded and the pellet was air-dried. To resolve the RNA, sterile nuclease-free water was added to the samples and the concentration was measured using Nanodrop spectrophotometer.

### Quantitative real-time PCR

Up to 1µg of RNA was transcribed into cDNA using the RevertAid™ First Strand cDNA synthesis kit (ThermoFisher, Waltham, USA) according to the manufacturer´s instructions. For the qPCR reaction, cDNA in a concentration of 5ng/µl was used and mixed with iTaq Universal SYBR^®^ Green Supermix (Bio-Rad Laboratories GmbH, Feldkirchen, Germany), UltraPure™ DEPC-treated Water (Invitrogen™ (ThermoFisher), Waltham, USA) and the respective forward and reverse primers. qPCR primers were purchased from Eurofins Genomics (Luxemburg, Luxemburg), and the primer sequences are shown in **Supplementary Table S4**. qPCR reactions were performed for 50 cycles with an initial activation phase for 2 min at 98°C, denaturation phase for 5 s at 95°C and an annealing and extension phase for 10 s at 60°C. Measurements were performed using the CFX-96 Real-Time PCR Detection system and data analysis were performed using the CXF Manager Software (Bio-Rad Laboratories GmbH, Feldkirchen, Germany). The relative expression level of specific transcripts was calculated using the 2^-ΔΔCT^ method for tissue analysis and the 2^-ΔCT^ method for PBMCs analysis.

### Protein isolation of lung tissue

To isolate the proteins from the tissue, a piece of tissue was placed in a lysis tube. 200 µl of lysis buffer (10 ml M-Per Mammalian Protein Extraction Reagent (Thermo Fisher Scientific, Waltham, MA, USA), one tablet Mini EDTA-free Protease-Inhibitor-Cocktail (Merck, Darmstadt, Germany) and one tablet PhosSTOP™ (Merck, Darmstadt, Germany) were added to the tissue pieces and digested in a homogenizer (SpeedMill PLUS, Analytic Jena, Jena, Germany) for 1 min. The lysis tubes were then centrifuged for 5 minat 3000 rpm and the supernatant was transferred to a new tube. The lysate was incubated on ice for 30 min and centrifuged for 5 minutes (2000 x g, 4°C). The supernatant was transferred again and centrifuged for 45 minat maximum speed (4°C). Subsequently, the supernatant was collected and the protein concentration was determined using the Bradford reagent. In order to determine the exact amount of protein, a standard series using BSA was done.

### Gel electrophoresis and Western blotting

Mini-PROTEAN TGX Stain-Free Precast Gels (Bio-Rad Laboratories GmbH, Feldkirchen, Germany) were used for the gel electrophoresis. Here, 20 µg of each sample were used in a final volume of 20 µl. For this purpose, the appropriate amount of protein was stocked up to a volume of 13 µl with DEPC-water, mixed with 2 µl dichlorodiphenyltrichloroethane (DDT) and 5 µl loading buffer (4x NuPage™ LDS-Buffer, Invitrogen (Thermo Fisher Scientific), Waltham, MA, USA) and heated at 95°C for 5 min. The samples were loaded onto a gel and run at 120 V. For subsequent western blotting, the gel was transferred to a PVDF membrane using the Trans-Blot Turbo Mini PVDF Transfer Pack (format 0.2 µm) and the Trans-Blot Turbo Transfer System (Bio-Rad Laboratories GmbH, Feldkirchen, Germany). The transfer was carried out for 7 min at 25 V and 2,5 A. Blocking and antibody staining for PU.1 was performed according to the manufacturer´s protocol (Cell Signaling Technology, Danvers, USA). For Western Blot analysis, total protein and specific bands were determined using Image Lab software (Bio-Rad Laboratories GmbH, Feldkirchen, Germany).

### Cell isolation of lung tissue

For the isolation of cells from human lung tissue, the tissue pieces were thoroughly minced with a scalpel and then digested overnight at 37°C with a collagenase-DNase mix (270U/ml collagenase; 150 µl DNaseI). The digested tissue was pressed through a 100 µm cell sieve into a sterile tube using a syringe plunger and rinsed with RPMI 1640 medium. The cells were centrifuged for 7 min at 300 x g (RT), resuspended in 10 ml ACK lysis buffer and immediately centrifuged again (5 min, 1500 rpm, 4°C). The pellet was resuspended in wash buffer (PBS-EDTA + 1% Pen/Strep) and centrifuged again (1500 rpm, 5 min, 4°C). The cells were resuspended in a second wash buffer (PBS-EDTA + 1% Pen/Strep + 5% FCS), centrifuged for 15 min (800 rpm, 4°C) with a low braking force (DEC:1), and resuspended in FACS-buffer for flow cytometric analysis.

### PBMC isolation

PBMC isolation was performed using Leucosep Tubes (Greiner Bio-One, Kremsmünster, Austria), according to the manufacturer´s protocol. The tubes were filled with 15 ml of BioColl separation medium (Bio&Sell, Feucht, Germany) and centrifuged (30 sec, 1000 x g, RT). The volume of EDTA blood was measured, diluted with the same volume of PBS, poured into a Leucosep tube, and centrifuged for 10 min at 1000 x g (RT) with the brakes switched off (DEC:0). After centrifugation, PBMCs were collected, washed with 10 ml of RPMI 1640 medium (anprotec, Bruckberg, Germany) and subsequently centrifuged (10 min, 250 x g, RT). The washing step was repeated twice with 5 ml of RPMI 1640 medium. To remove the remaining red blood cells, the pellet was resuspended in 5 ml of ACK-Lysing Buffer and immediately centrifuged for 5 minutes (1500 rpm, 4°C). In the final step, PBMCs were resuspended in 5 ml of RPMI 1640 medium and counted. For subsequent RNA isolation, some of the PBMCs were stored in Qiazol Lysis^®^ Reagent (QIAGEN, Hilden, Germany).

### Flow cytometry

For intracellular flow cytometric analysis between 500.000 and 1 million cells were stained in a 96 well U-bottom plate (Thermo Fisher, Waltham, USA). First, the cells were incubated with 50µl of Zombie Aqua™ dye (1:500 dilution in PBS; BioLegend, San Diego, CA, USA) for 15 min at RT. After that, 150µl of FACS-buffer was added, and the cells were centrifuged for 1 min (2000 rpm, RT). Following another wash step with 150µl FACS-buffer and centrifugation, cells were resuspended in 50µl Human TruStain FcX™ (BioLegend, San Diego, CA, USA) and incubated for 10 min at RT. After washing the cells again with 150µl of FACS-buffer, the cells were centrifuged and resuspended in 50µl of the antibody mix for surface staining and incubated for 15 min at 4°C. To perform intracellular staining, cells were washed after surface staining, resuspended in 100µl of FoxP3 Fixation/Permeabilization reagent (Thermo Fisher, Waltham, USA) and incubated for 30 min at RT. Afterwards, the cells were washed twice with 200µl of permeabilization buffer (1 part Thermo Fisher Scientific Permeabilization Buffer (10X), and 9 parts Millipore-H2O). Subsequently, 50µl of the intracellular antibody mix was added and the cells were incubated for 30 min at RT. After a final wash step with 200µl of permeabilization buffer and centrifugation, cells were resuspended in 100µl FACS-buffer and analyzed using the BD FACSymphony™ A1 Cell Analyzer (BD Biosciences, Heidelberg, Germany). Unstained controls were included in all experiments and processed in parallel using the same intracellular staining protocol. The antibodies used are listed in **Supplementary Table S3**. Data sets were analyzed using Kaluza Flow Cytometry Software 2.1 (Beckman Coulter Inc., California, USA).

### Multiplex assay

Serum from NSCLC patients and healthy controls was analyzed for cytokines using the LEGENDplex™ Human Inflammation Panel 1 (13-plex) (BioLegend, San Diego, USA). The assay was performed using 96 Well U-Bottom plates (Thermo Fisher, Waltham, USA) according to the manufacturer´s protocol. For data acquisition, the beads were resuspended in 100µl of 1x wash buffer and measured using the BD FACSymphony™ A1 Cell Analyzer (BD Biosciences, Heidelberg, Germany). Data sets were analyzed using the Kaluza Flow Cytometry Software 2.1 (Beckman Coulter Inc., California, USA).

### Statistics

Statistical analyses were performed using GraphPad Prism 9 software to obtain the significance levels (*p < 0.05; **p < 0.01; ***p < 0.001; ****p < 0.0001). Data were imported into column statistics and then analyzed for normal distribution using the Shapiro-Wilk test and D´Agostino-Pearson test. For normally distributed values, one-way analysis of variance (ANOVA) multiple comparisons was performed. For non-normal distributions or n<5 the Kruskal-Wallis test was used. For two independent groups with normal distribution, the unpaired t-test was used. For non-normal distributions, the Mann-Whitney test was used. Grouped data were analyzed using the two-way ANOVA test. The correlations were examined using linear regression curves. Two-tailed Pearson correlation analysis was performed to obtain the r and p values. Survival data are represented in Kaplan–Meier survival curves and the log rank test was used to compute p values. Data are presented as the mean ± SEM. The number of samples analyzed for each experiment is shown in the figure legends.

## Results

### PU.1 mRNA expression is increased in lung tumor tissue

**Figure 1:**
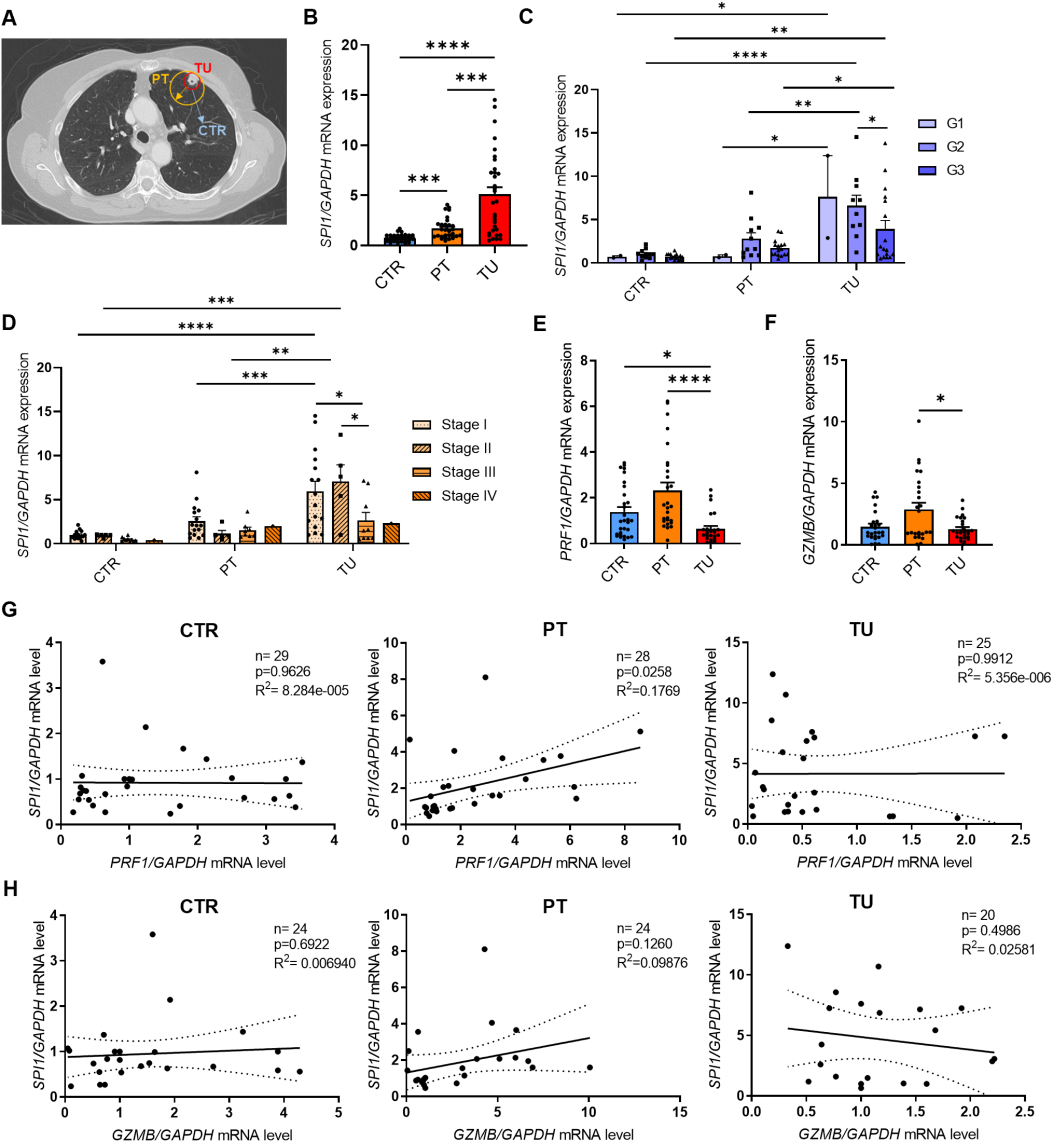
*SPI1* mRNA expression is induced in the tumoral region of the lung from patients with NSCLC. The expression of PU.1 mRNA was analyzed in the tumoral region (TU), peritumoral region (PT) and a tumor-free control region (CTR) of the lung from patients with NSCLC. **(A)** Representative lung-CT image from a NSCLC patient with indicated tissue areas (CTR,PT,TU). Lung samples were dissected from the tumor area (TU), the peri-tumoral area (PT) surrounding the tumor and from the control area (CTR), which is at least 5 centimeters away from the tumor border. **(B)** *SPI1* mRNA level from total lung cells was normalized on *GAPDH* mRNA level (nCTR=31; nPT=29; nTU=32). **(C)** *SPI1/GAPDH* mRNA levels after classification by grading of tumor cell differentiation (nG1 = 2; nG2 = 10-11; nG3 = 16-17) and **(D)** TNM-stage (nStage I=15-16; nStage II=5; nStage III= 8-10; nStage IV= 1). **(E)** *PRF1* (nCTR=29; nPT=27; nTU=25) and **(F)** *GZMB* mRNA level from total lung cells was normalized on *GAPDH* mRNA level (nCTR=25; nPT=25; nTU=26 **(G)** *PRF1/GAPDH* mRNA expression level and **(H)** *GZMB/GAPDH* mRNA expression level was correlated with *SPI1/GAPDH* mRNA expression level in the lung control region, peritumoral and tumoral region of NSCLC patients. N values are given per group. Bar charts indicate mean values +/- s.e.m. using 2-way-ANOVA **(C, D)**, Kruskall-Wallist test **(E)** or Brown-Forsythe and Welch ANOVA test **(B, F)**. *p ≤ 0.05; **p ≤ 0.01; ***p ≤ 0.001; ****p ≤ 0.0001.

In this study, we enrolled and performed analysis of 61 patients diagnosed with NSCLC (**Supplementary Table S1**) and 34 healthy control subjects (**Supplementary Table S2**). The clinical data of all participants are summarized in **Table 1**. To investigate the role of the transcription factor PU.1 in NSCLC, we collected post-surgery lung tissue samples from the tumoral region (TU), peritumoral region (PT) and tumor-free control region (CTR) of NSCLC patients (**Figure 1A** **).** First, we analyzed the expression of PU.1 (*SPI1*) on mRNA level in the different lung tissue regions. This analysis showed induction of *SPI1* in relation to *GAPDH* mRNA expression in the peritumoral and tumoral region of the lung compared to that in the control region (**Figure 1B** **).** Induced *SPI1* mRNA level in the tumoral region was also confirmed in relation to *HPRT* mRNA (**Supplementary Figure S1A**). To determine whether the increased expression was dependent on the progression of NSCLC, we grouped the patients according to tumor grading (**Figure 1C**) and tumor stage (**Figure 1D**). Here, we found a significant increase in *SPI1* mRNA expression in the tumoral region of the lung of patients with lung tumor gradings G2 and G3, as well as in patients with TNM-stage I and by tendency in patients with TNM-stage II. To understand the role of increased PU.1 in the tumoral region, we next investigated the connection between PU.1 and various genes involved in cytotoxicity, such as perforin (*PRF1*) and granzyme B (*GZMB*). Therefore, we measured the mRNA expression of these genes (**Figures 1E, F**) by qPCR and found both mRNA levels increased by tendency in the peritumoral region, as well as significantly decreased perforin and granzyme B expression in the tumoral region of the lung compared to the peritumoral region. Next, we correlated the mRNA expression levels of perforin (**Figure 1G**) and granzyme B (**Figure 1H**) with *SPI1* mRNA expression and discovered a positive correlation between *SPI1* and *PRF1* in the peritumoral region of patients with NSCLC.

**Table 1:** Clinical characteristics of lung cancer and control patients.

Group	Characteristic		n	Percentage
NSCLC patients	Age (years)		68,59 (± 8,38)	
	Gender	Male	32	52,5%
		Female	29	47,5%
	Histology	ADC	45	73,8%
		SCC	13	21,3%
		ADC-SCC	3	4,9%
	Grading	G1	3	4,9%
		G2	23	37,7%
		G3	33	54,1%
		unknown	2	3,3%
	Pathologic stage	I	35	57,4%
		II	9	14,8%
		III	15	24,6%
		IV	2	3,3%
	Smoking history	Non-Smoker	8	13,1%
		Smoker (active)	35	57,4%
		Former Smoker	18	29,5%
Healthy controls	Age (years)		45,82 (± 13,85)	
	Gender	Male	19	52,25%
		Female	15	47,5%
	Smoking history	Non-Smoker	23	67,6%
		Smoker (active)	10	29,4%
		Former Smoker	1	2,9%

### Intra-tumoral NK cells express induced PU.1 levels in the lung

**Figure 2:**
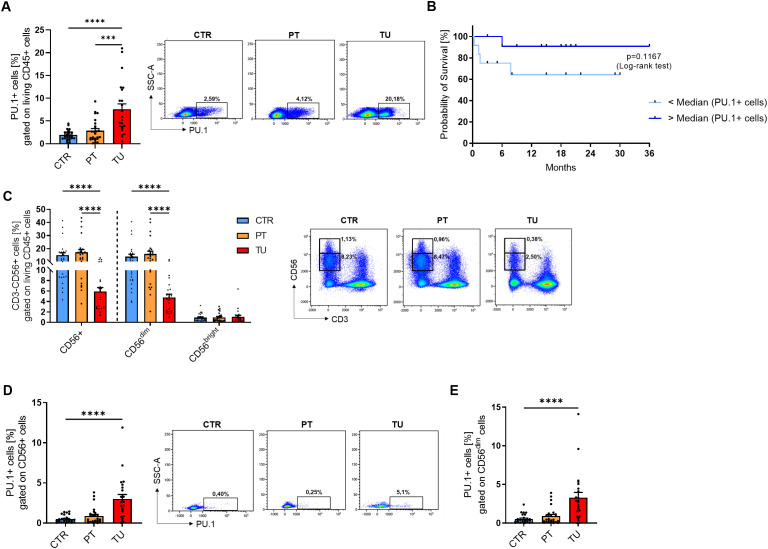
PU.1 expression is induced in NK cells from the tumoral region of the lung from patients with NSCLC. Cells were isolated from the tumoral region (TU), peritumoral region (PT) and control region (CTR) of the lung from patients with NSCLC and analyzed by flow cytometry. **(A)** Flow cytometry analysis of PU.1+ cells (%) gated on living CD45+ cells of cells isolated from CTR, PT and TU region of NSCLC patients (nCTR=25; nPT=25; nTU=23). **(B)** The median value of PU.1+CD45+ cells (%) in the tumoral region of NSCLC patients was calculated and patients were grouped according to this value (amount of PU.1+CD45+ cells was lower (n=12) or higher (n=12) than the median value). On this basis, a survival curve was created. **(C)** Quantification of CD3-CD56+ NK cells (%) gated on living CD45+ cells isolated from CTR, PT and TU region of NSCLC patients. CD56+ cells were additionally separated into CD56^dim^ and CD56^bright^ subpopultions (nCTR=25; nPT=25; nTU=23). **(D)** Quantification of PU.1+ cells (%) gated on CD56+ total cells and **(E)** CD56^dim^ cells (nCTR=26; nPT=26; nTU=23). N values are given per group. Bar charts indicate mean values +/- s.e.m. using Kruskall-Wallist test **(A, D, E)** or 2-way-ANOVA test **(C)**. ***p ≤ 0.001; ****p ≤ 0.0001.

We next analyzed PU.1 at the protein level and started with western blot analysis (**Supplementary Figure S1B**). Here, we also detected upregulation of PU.1 in the tumoral region of the lung in patients with NSCLC. To determine in which cell population PU.1 was upregulated, we isolated cells from the different lung tissue regions and analyzed them by flow cytometry. We started with an analysis of PU.1 in the total CD45+ leucocytes (**Figure 2A**) and confirmed our western blot results, as PU.1+ CD45+ cells were significantly increased in the lung tumor tissue. We then divided the patients included in the flow cytometric analysis according to their tumor grading and found PU.1 increased in the tumor regions of both gradings G2 and G3 (**Supplementary Figure 2A**). At this point, we asked about the influence of increased PU.1 expression on patients´ overall survival. Thus, we calculated the median percentage of PU.1+ CD45+ cells in the tumoral region of our cohort and grouped the patients accordingly. Patients with values lower than the median PU.1 expression were grouped, as were patients with a value higher than the median expression of PU.1. On this basis, a Kaplan-Meier survival curve was created (**Figure 2B**), which showed better overall survival for patients with a higher amount of PU.1+ CD45+ cells in the tumoral region. To explain this observation, we examined several populations with NK cells in the focus. NK cells were defined as living (Zombie-) CD45+ CD3- and CD56+ cells and were further divided into two major subsets based on their expression levels of CD56: CD56^dim^ and CD56^bright^ (**Figure 2C**). Here, the population of total CD56+ NK cells, as well as the CD56^dim^ subpopulation, was found to be significantly decreased in the tumoral region as compared to the peritumoral and control region of the lung. Simultaneously, there was no difference in the number of CD56^bright^ cells between the three regions of the lung, indicating that the CD56^dim^ subset was more affected by the tumor cells than the CD56^bright^ NK cells. Therefore, we analyzed the individual NK cell populations for PU.1 expression and detected a significant increase of this transcription factor in total tumoral CD56+ NK cell (**Figure 2D**) and CD56^dim^ NK cell (**Figure 2E**) fractions in comparison to the respective peritumoral and control area. Regarding the CD56^bright^ NK cells (**Supplementary Figure 2B** **),** not all patients showed an expression of PU.1 in this cell population in the lung. However, in the majority of patients PU.1 was induced here as well.

### Induced expression of PU.1 in peripheral blood from NSCLC patients as compared to healthy control subjects

**Figure 3:**
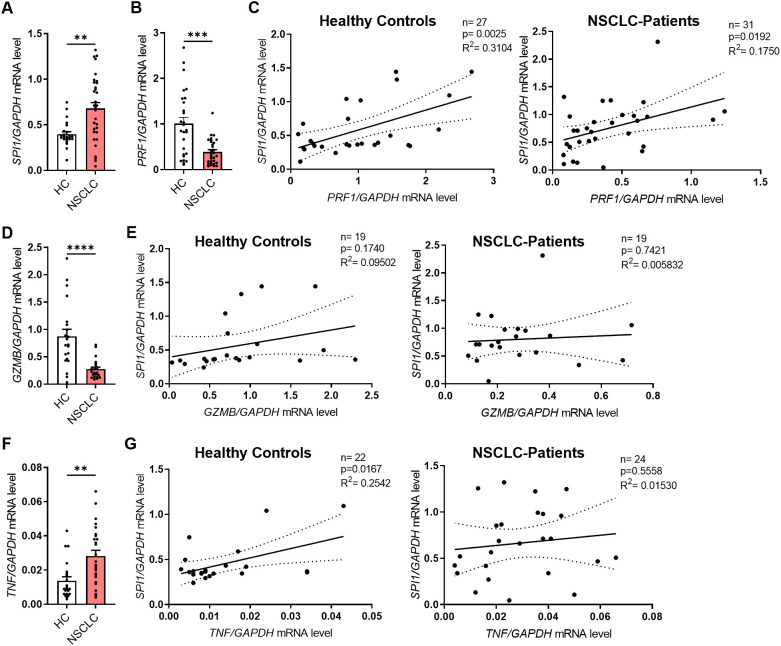
*SPI1* mRNA expression is induced in PBMCs from NSCLC patients and correlates with cytotoxic function. Peripheral blood mononuclear cells (PBMCs) were isolated from blood of healthy controls (HC) and NSCLC patients. **(A)** *SPI1* mRNA level (nHC=24; nNSCLC=32) and **(B)** *PRF1* mRNA level (nHC=27; nNSCLC=31) from PBMCs of healthy controls and NSCLC patients was normalized on *GAPDH* mRNA level. **(C)** Corrleation of *PRF1/GAPDH* mRNA levels with *SPI1/GAPDH* mRNA levels of healthy controls (n=27) and NSCLC patients (n=31). **(D)** *GZMB* mRNA level was normalized on *GAPDH* mRNA level (nHC=21; nNSCLC=22) and **(E)** correlated with *SPI1*/*GAPDH* mRNA levels. **(F)** *TNFa* mRNA level was normalized on *GAPDH* mRNA level (nHC=21; nNSCLC=22) and **(G)** correlated with *SPI1*/*GAPDH* mRNA levels. N values are given per group. Bar charts indicate mean values +/- s.e.m. using Mann-Whitney test. **p ≤ 0.01; ***p ≤ 0.001; ****p ≤ 0.0001.

In addition to lung tissue material from patients with NSCLC, we analyzed blood from a subgroup of the patient cohort and healthy control subjects. In this part of the study, we isolated peripheral blood mononuclear cells (PBMCs) and serum from the blood. First, we isolated RNA from fresh PBMCs and performed qPCR analysis for *SPI1* and several cytokine genes. Consistent with our initial results on the lung tissue region, *SPI1* mRNA level was significantly higher in PBMCs from patients (**Figure 3A**, **Supplementary Figure 3A**) than in those from healthy controls. Although we found that both perforin (**Figure 3B**) and granzyme B (**Figure 3D**) were significantly less expressed at mRNA level in patients with lung cancer, *PRF1* (**Figure 3C**), but not *GRZMB* (**Figure 3E** **),** positively correlated with *SPI1* mRNA expression in both cohorts. We also analyzed the correlation with other granzymes, A and M at mRNA level. The analysis showed that both granzymes were expressed at lower levels in the peripheral blood of NSCLC patients (**Supplementary Figures 3B, D**), but equally to *GZMB* there was no correlation with *SPI1* mRNA expression (**Supplementary Figures 3C, E**). Since TNFα is one of the cytokines secreted by NK cells, we also analyzed *TNF* mRNA expression. We detected higher *TNF* mRNA levels in PBMCs from patients with lung cancer compared to healthy control subjects (**Figure 3F** **),** suggesting enhanced inflammatory activity in the peripheral blood and indicating a systemic immune response associated with lung cancer. The positive correlation between *SPI1* and *TNF* mRNA observed in healthy controls, but absent in patients (**Figure 3G**), may indicate a disrupted or altered regulatory relationship between PU.1 and TNF in NSCLC.

### High expression of PU.1 in PBMCs is connected to recurrence-free and metastasis-free survival

**Figure 4:**
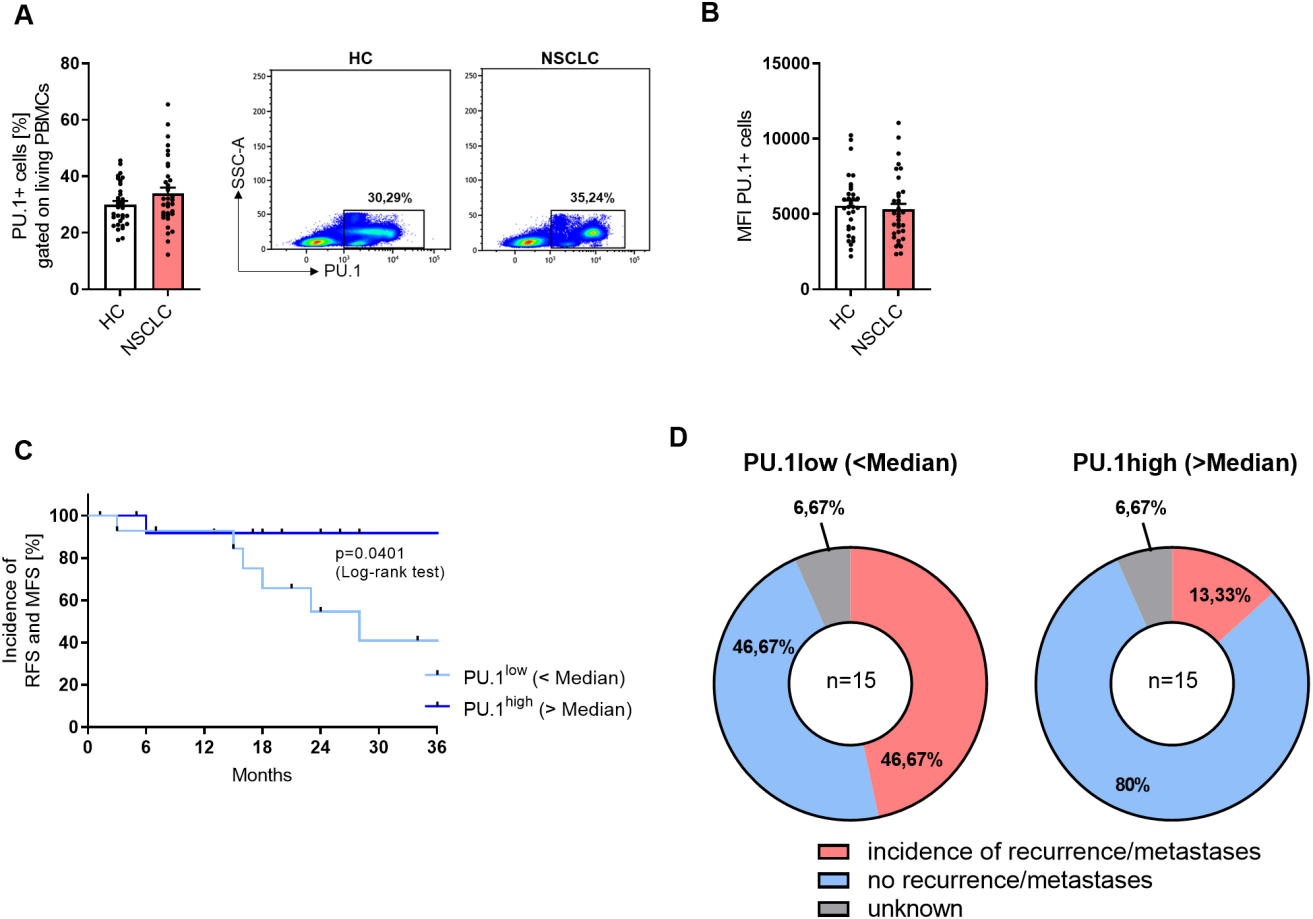
NSCLC patients with a high expression of PU.1 in the PBMCs show increased RFS and MFS. Peripheral blood mononuclear cells (PBMCs) were isolated from blood of healthy controls (HC) and NSCLC patients. **(A)** Flow cytometry analysis of PU.1+ cells in relation to total living PBMCs [%] (nHC=32; nNSCLC=34) **(B)** and Mean Fluorescene Intensity (MFI). **(C)** The median value of PU.1+ PBMCs [%] of the NSCLC cohort was calculated and patients were grouped according to this value (amount of PU.1+PBMCs was lower or higher than the median value). On this basis, a Kaplan-Meier curve for recurrence-free survival (RFS) and metastases-free survival (MFS) was calculated. **(D)** Distribution of patients regarding the incidence of recurrences or metastases within the PU.1^low^ (n=15) and PU.1^high^ (n=15) group. N values are given per group. Bar charts indicate mean values +/- s.e.m.

In the next step, we analyzed freshly isolated PBMCs by flow cytometry. The gating strategy is described in detail in **Supplementary Figure 4**. When we gated the total PBMCs, no difference in the PU.1 expression between the cohorts was detectable (**Figures 4A, B**). However, when we calculated the median PU.1 expression level and grouped the NSCLC cohort based on the median value in a PU.1^low^ and a PU.1^high^ group, we found that the PU.1^high^ group showed a significantly increased recurrence-free survival (RFS) and metastases-free survival (MFS) (**Figure 4C** **).** While approximately 47% in the PU.1^low^ group were still metastases and recurrence-free 36 months after surgery, at the same time point, in the PU.1^high^ group the proportion of metastases and recurrence free patients accounted for 80% (**Figure 4D**).

### Peripheral NK cells from lung cancer patients with increased PU.1 expression show lower cytotoxic potential

**Figure 5:**
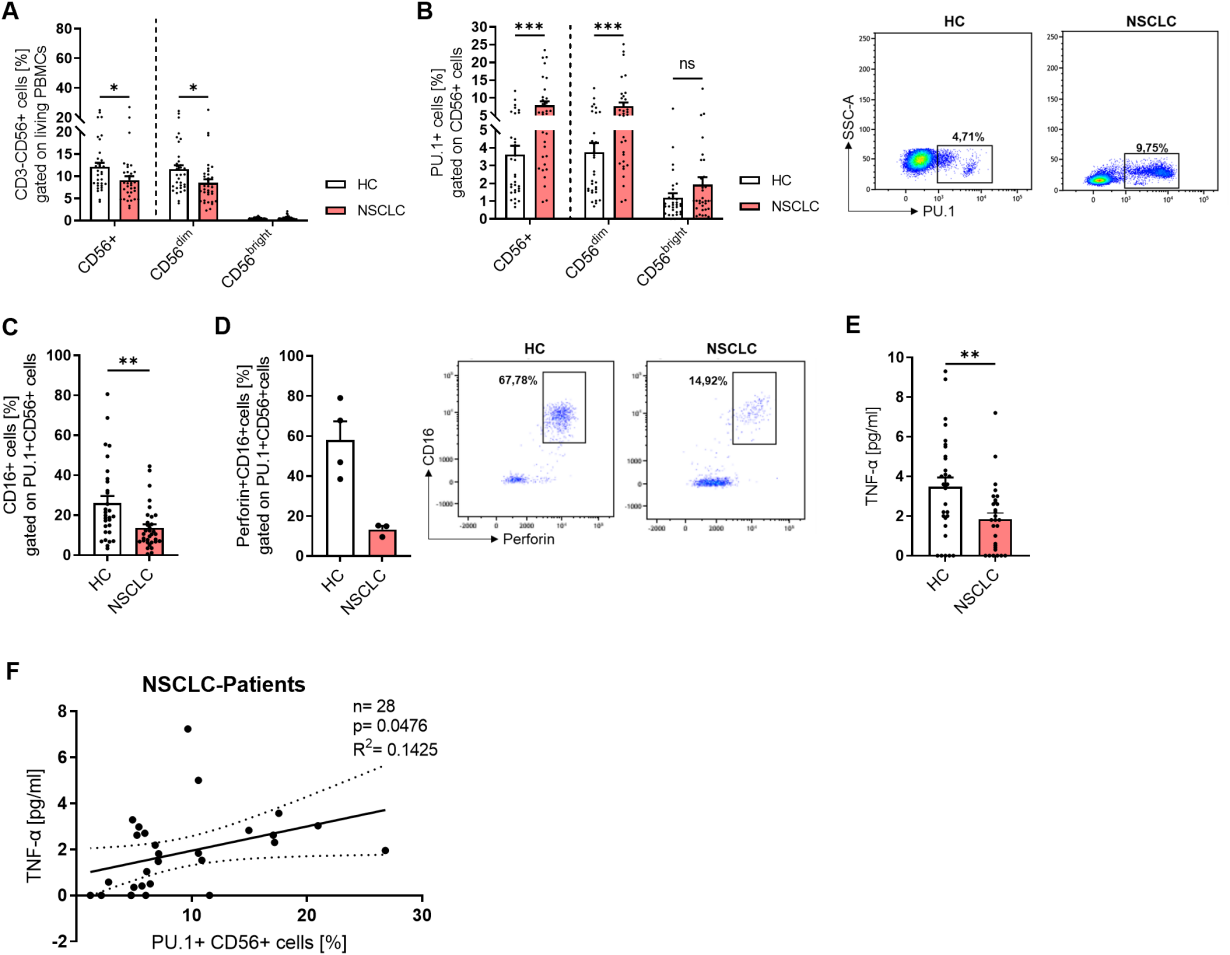
PU.1 expression is induced in peripheral NK cells from NSCLC patients. Peripheral blood mononuclear cells (PBMCs) were isolated from blood of healthy controls (HC) and NSCLC patients and analyzed by flow cytometry. **(A)** Quantification of CD3-CD56+ cells (%) gated on living PBMCs and analysis of CD56+ total cells as well as CD56^dim^ and CD56^bright^ subpopulations (nHC=33; nNSCLC=34). **(B)** Quantification of PU.1+ cells (%) gated on CD56+ total cells and CD56^dim^/CD56^bright^ subpopulations (nHC=32; nNSCLC=33). **(C)** Analysis of CD16 expression (nHC=30; nNSCLC=32) and **(D)** CD16+Perforin+ cells (nHC=4; nNSCLC=3) gated on PU.1+ CD56+ cells. **(E)** TNF-α levels [pg/ml] were measured in serum from healthy controls (n=30) and NSCLC patients (n=29) and **(F)** correlated with PU.1+ CD56+ cells (%) in NSCLC patients. N values are given per group. Bar charts indicate mean values +/- s.e.m. using 2-way-ANOVA test **(A, B)** or Mann-Whitney test **(C, E)**. *p ≤ 0.05; **p ≤ 0.001; ***p ≤ 0.001.

In accordance with the lung tissue analysis from our NSCLC cohort, we analyzed the NK cell populations in the PBMCs (**Figure 5A**). While total CD56+ NK cells and CD56^dim^ cells were significantly decreased in PBMCs from lung cancer patients, the CD56^bright^ subset was equally present at low levels in both cohorts. We gated NK cells that expressed PU.1 (**Figure 5B**), and detected an increased expression of the transcription factor in total CD56+ NK cells and the CD56^dim^ subset. Since CD16 (FcγRIII) plays a key role in antibody-dependent cell-mediated cytotoxicity (ADCC), we next examined its expression in PU.1^+^CD56^+^ NK cells to assess potential alterations in cytotoxic function. We analyzed CD16 alone (**Figure 5C**) and in combination with perforin expression (**Figure 5D**) in the population of PU.1+ NK cells, and discovered a significantly decreased expression of CD16 and a decrease by trend of CD16+Perforin+ cells in the NSCLC cohort. We wondered whether the cytokines secreted by NK cells, such as IFNγ, were affected by the expression of PU.1. Thus, we measured IFNγ serum levels (**Supplementary Figure 5A**), but did not find any differences between the two cohorts. As the transcription factor T-bet is known to be highly correlated with IFNγ expression in NK cells, we included this marker in our analysis and identified a population of PU.1+T-bet+ CD56+ cells. However, this population was equally expressed in both cohorts (**Supplementary Figure 5B**). Next, we asked for a relation between PU.1 and TNFα. Since we already found a correlation between *TNF* and *SPI1* mRNA expression levels, we measured TNFα in the serum from both cohorts (**Figure 5E**) and found significantly less TNFα in the serum of NSCLC patients, which could reflect a diminished systemic inflammatory state that might affect immune surveillance in lung cancer patients. Moreover, we found that serum TNFα levels were positively correlated with the proportion of PU.1+CD56+ cells in patients with lung cancer (**Figure 5F**), but not in healthy subjects (**Supplementary Figure 5C**), suggesting a potential link between PU.1- expressing NK cells and circulating TNFα in the context of lung cancer.

## Discussion

Since lung cancer does not usually cause symptoms at the onset of the disease, it is often diagnosed and treated at an advanced stage of the tumor, which impedes successful therapy. At an early tumor stage, treatment most commonly involves surgical removal of the primary tumor followed by chemotherapy, radiotherapy or immunotherapy (23). Bronchial carcinomas are solid tumors that develop directly in tissues. They are initially localized, but can spread through the body by shedding tumor cells and thus also form metastases in other organs not affected by the primary tumor, which makes successful treatment more difficult. In addition to the cancer cells themselves, the tumor microenvironment in solid tumors consists of non-malignant cells such as fibroblasts, epithelial cells and immune cells (24). Immune cells in the vicinity of the tumor are mainly composed of T cells, B cells, NK cells, dendritic cells, monocytes and macrophages. These different cell types can either play an anti-tumoral role or even promote tumor growth by inhibiting the immune response.

Previous studies have shown that point mutations or deletions in PU.1 can lead to the development of acute myeloid leukemia (AML) (18). Furthermore, accumulation of PU.1+ Th9 cells was observed in murine models of colon carcinoma. Studies have indicated the absence of IL-9 or PU.1 inactivation in T cells resulted in decreased tumor growth, implicating a pro-tumoral role of PU.1+ Th9 cells (20, 21). These and other results showed that PU.1 could be a meaningful factor in the diagnosis, prognosis or treatment of patients with cancer. However, the exact function of PU.1 in different cancer types and cell populations is controversial.

Studies often focus only on the tumor area itself, comparing it to adjacent healthy tissue, while the tumor margins are rarely or insufficiently analyzed. Nevertheless, the peritumoral tissue surrounding the tumor may also contain tumor cells which, if not sufficiently removed, can contribute to tumor recurrence. The region that was already described in the 1980s as a “reactive zone” (25) can influence tumor growth and the formation of metastases. This is caused by cells of the peritumoral region secreting growth factors and cytokines, which in turn can promote hypoxia and angiogenesis (13). Modifications within the peritumoral environment can also influence the prognosis of patients with malignant tumors (26). Therefore, in this study, the peritumoral region was additionally examined after surgical removal of the lung tumor along with a control region that was free of tumor cells. Analysis of *SPI1* mRNA expression, which encodes the transcription factor PU.1, showed a significantly higher expression in the peritumoral region compared to the control region of the lung, which confirms an inflammatory environment in this region. Notably, SPI1 expression was even further elevated in the tumor region, suggesting that PU.1 may also play a key role in the immunological or pathological processes within lung tumors. Although public RNA-seq datasets (e.g., TCGA) show reduced *SPI1* expression in NSCLC tumor tissue compared to normal tissue, these data represent bulk transcriptomes and may not reflect immune-cell–specific protein expression and function. In our cohort, the greatest induction of *SPI1* was observed in both the peritumoral and the tumoral region in patients with grade 2 and stage I tumors. Based on the data available here, it is not possible to make a clear statement about the situation of patients with tumor grade 1 and stage IV due to the insufficient number of patients in this disease stage. However, since patients in stage III already showed lower expression than patients in stage II, it is possible that this is similar for stage IV and PU.1 was downregulated with increasing tumor disease severity. The increased expression of SPI1 (PU.1) in the peritumoral and tumor region in patients with early tumor stages could indicate an active immune response at the early to intermediate course of the tumor disease. A higher expression could therefore imply an increased infiltration of PU.1+ immune cells or an activated immune environment. However, as the disease progresses, particularly from stage III onwards, SPI1 expression appears to decrease. This reduction could be an expression of increasing immunosuppression in the tumor microenvironment or a tumor-mediated mechanism of immune escape. In advanced stages, the reduced expression could thus indicate a functional depletion or repression of PU.1+ immune cells, possibly due to immunomodulatory processes such as the recruitment of regulatory cells or the upregulation of inhibitory checkpoints.

Overall, the available data indicate a stage- and grade-dependent regulation of SPI1 in the tumor microenvironment, which could correlate with the immunological status and progression of the tumor disease.

A similar trend was observed at the protein level using western blotting, showing an apparent increase of PU.1 in the tumor region. This was further supported by flow cytometric analysis, which demonstrated significantly higher PU.1 expression in this region. PU.1+ cells could accumulate there and potentially strengthen the anti-tumoral immune response. In a recent study, Liu et al. identified PU.1 as a tumor suppressor in NSCLC (27), which could be confirmed by our data. However, a detailed analysis of the lymphocytes in the lung is necessary to define the significance of the high expression of PU.1 more precisely. Although the survival curves determined from the median PU.1 expression - where patients with a high PU.1 expression in the tumoral region survived longer - can be seen as an indication of a protective role of PU.1 and again align with the results of Liu et al. (27), it must be kept in mind that this is an ongoing and not a retrospective study. Although, it is currently not possible to observe all patients over the same period of time, we additionally found a connection between a high expression of PU.1 in the blood of patients with NSCLC and an increased recurrence and metastases-free survival which emphasized the protective role of a high PU.1 expression in the periphery. The period of time patients with a high expression of PU.1 survived without the incidence of metastases or recurrence was longer compared to those with a low PU.1 expression. Furthermore, the proportion of patients suffering from recurrence was clearly lower in the PU.1^high^ group, indicating that PU.1 expression in the blood could be used as a predictive marker for the incidence of recurrence and metastases in lung cancer.

NK cells can promote tumor defense by recruiting and activating other immune cells and exerting cytotoxic functions. Functional defects and reduced activity of NK cells in the peripheral blood are associated with a higher incidence of carcinomas (28, 29). A correlation between the number of tumor-infiltrating NK cells and the prognosis of various types of cancer, including lung cancer, has also been established (29–32).The results of this study showed that cytotoxic CD56^dim^ accounted for a greater proportion of NK cells in the lung and that the proportion of total NK cells in the tumor region decreased significantly, suggesting a generally reduced tumor defense. In addition to reduced proliferation, functional defects in the CD56^dim^ population may also result in loss of IFNγ production and lack of activation (33). Since the peritumoral region was analyzed in this study and the ratio of NK cells was similar to that of the control region, it is possible that the peritumorally accumulated cells had already acquired a defect in their migration ability and were thus unable to enter the tumor and exert their effector functions. One reason for this could be that the peritumoral region cannot be seen as healthy tissue but is definitely influenced by tumor-associated chemokines (31). However, intra-tumoral NK cells have a different phenotype and functional impairment compared to NK cells located in healthy tissues. This has been described for patients with lung cancer (31, 32), prostate cancer (34), breast cancer (35) and gastrointestinal tract tumors (36). NK cell dysfunction has been attributed to cytokines secreted by MDSCs and macrophages, such as TGFβ and IL-10 (32). Furthermore, reduced anti-tumor function was associated with decreased NK cell viability caused by the downregulation of activating ligands and an immunosuppressive tumor environment (32). Our analysis of tumoral and non-tumoral NK cells of the lung demonstrated not only a reduced number of NK cells in the tumor, but also a significantly increased expression of PU.1 in this region. As PU.1 mRNA expression was significantly correlating with perforin mRNA expression and NK cells are closely associated with perforin secretion (37, 38), these results imply that a higher expression of PU.1 in NK cells could lead to an increased cytotoxic potential of the cells. However, the anti-tumoral effects seem to be blocked either in the process of protein translation or by the strong influence of the tumor microenvironment. Either way, the impact of PU.1+ NK cells on the effector functions of NK cells must be examined in more detail.

Our data demonstrate that *PRF1* and *GZMB* expression levels are elevated in the peritumoral region compared to the tumor core, suggesting a spatial heterogeneity in cytotoxic immune activity. This pattern may reflect the presence of functionally active cytotoxic lymphocytes at the tumor margin, whereas the tumor core exhibits features of immune suppression, possibly due to altered cytokine profiles, stromal factors, or immune cell exhaustion. The elevated expression of PU.1 in the peritumoral region raises the possibility that PU.1 may contribute to sustaining cytotoxic potential in this compartment. While our data indicate a positive correlation between PU.1 and PRF1 mRNA expression in certain contexts and PU.1 is known to control the differentiation and activation of various immune cells, including NK and T cells (17, 39), current literature has not yet identified *PRF1* as direct PU.1 target. Therefore, the observed expression patterns could reflect an enrichment of PU.1-expressing cytotoxic lymphocyte subsets or indirect transcriptional effects. Further mechanistic studies, such as PU.1 overexpression or knockdown in relevant immune cell types, would be necessary to determine whether PU.1 functionally modulates perforin expression.

In PBMCs, the transcription factor PU.1 was examined under healthy conditions and upon the development of lung tumor. The development and differentiation of NK cells are regulated by various transcription factors and their interactions with each other. Several processes, such as cellular activation, differentiation and oncogenesis, are controlled by transcription factors of the ETS family (40–42), such as PU.1 or ETS1. PU.1 is already expressed in common lymphoid progenitor cells and regulates the transition to NK progenitor cells. In addition, PU.1 is expressed in both immature and mature NK cells (43). In the absence of PU.1, other transcription factors of the ETS-family are upregulated in NK cells, suggesting that they can compensate for the absence of PU.1 to maintain fundamental functions. Although NK cells were not examined as an isolated population for mRNA expression analysis, the expression of SPI1 was significantly increased in the total PBMCs of NSCLC patients. Even though PU.1 is also associated with IFNγ production and further cytotoxic functions of NK cells (44), we could not detect a connection between PU.1 and IFNγ in our cohort. However, although we found a lower level of TNFα in the serum of NSCLC patients, TNFα serum levels positively correlated with the proportion of PU.1+ NK cells in the blood. These data indicate a higher potential for this population to secrete inflammatory cytokines, as TNFα leads to enhanced NK cell functions (45). Moreover, we found that PU.1 directly correlated with perforin mRNA levels. Perforin forms a channel in the cell membrane of the target cell, allowing granzymes to enter the cell and trigger apoptosis. Like IFNγ, perforin is considered to act as an anti-cancer mediator that can control the growth and spread of tumors. However, reduced expression of perforin was found in T cells, NK cells and NKT-like cells in the tumor tissue of patients with lung cancer (46), which was confirmed in total PBMCs and PU.1+ NK cells. Similarly, the mRNA expression levels of granzyme A, B and M were downregulated in PBMCs from lung cancer patients, supporting a reduced cytotoxic capacity of peripheral blood cells in the context of cancer (47–49). In contrast to perforin, there was no relation between any of the granzymes and PU.1. Since the gene expression analyses revealed an initial indication that PU.1 could be related to the cytotoxic function of PBMCs, specific cell types were examined in more detail as part of the flow cytometric analysis. Total NK cells were less abundant in the blood of patients. After dividing the total NK cells into cytotoxic CD56^dim^ and cytolytic CD56^bright^ NK cells, this difference could be attributed to the CD56^dim^ NK cells. As other studies have already shown, this was to be expected, since CD56^dim^ cells make up the majority of NK cells, but can exhibit a defect in proliferation in the tumor environment (33). PU.1 was notably increased in NK cells from NSCLC patients with the CD56^dim^ subset being more affected than the CD56^bright^ subset. Once we characterized the function of PU.1+ NK cells in more detail, we discovered reduced expression of FcγRIII (CD16) in PU.1+ NK cells of the patients, which is associated with reduced cytotoxic function and a partial reduction of antibody-dependent cell-mediated cytotoxicity (ADCC) (50).

One limitation of our study is the mismatch in smoking history and age distribution between NSCLC patients and healthy controls in the PBMC-based analyses. Due to the fundamental role of smoking as the major risk factor for lung cancer, the majority of NSCLC patients in our cohort were current or former smokers. In contrast, many healthy individuals of comparable age are lifelong non-smokers. Recruiting age-matched healthy adults with no history of cancer or chronic respiratory diseases, who are nevertheless current or former smokers, poses a substantial challenge. As a result, the observed differences in PBMC gene expression may, in part, reflect differences in smoking status in combination with disease-specific effects. Thus, these findings require further validation in a more precisely matched control cohort. Overall, the transcription factor PU.1 appears to have a predominantly protective role in non-small cell lung cancer. However, the results of this study showed a possible dependence of the expression level on the severity of the disease. Generally, a high expression of PU.1 has been shown to be beneficial for the overall and metastases and recurrence-free survival. To further elucidate the protective mechanism of PU.1, or to maintain it even in advanced tumor stages, further studies are needed.

## Data Availability

The original contributions presented in the study are included in the article/**Supplementary Material**. Further inquiries can be directed to the corresponding author.
