# Supplementary material for: PU.1 upregulation is linked to improved prognosis in non-small cell lung cancer
Source: Front Immunol. 2025 Aug 15;16:1604237. doi: 10.3389/fimmu.2025.1604237 (PMC12395450; doi:10.3389/fimmu.2025.1604237)
Supplement: Supplementary file 1 [file DataSheet1.pdf]

Supplementary data

PU.1 upregulation is linked to improved prognosis in Non-Small Cell Lung Cancer

Katja Hohenberger[1], Denis I. Trufa[2], Arndt Hartmann[3], Susanne Mittler[1], Sonja Trump[1], Horia Sirbu[2,4,5] and Susetta Finotto[1,4,5,6*]

[1] Department of Molecular Pneumology, Friedrich-Alexander-Universität (FAU) Erlangen-Nürnberg, Universitätsklinikum Erlangen, Erlangen, Germany

[2] Department of Thoracic Surgery, Friedrich-Alexander-Universität (FAU) Erlangen-Nürnberg, Universitätsklinikum Erlangen, Erlangen, Germany

[3] Institute of Pathology, Friedrich-Alexander-Universität (FAU) Erlangen-Nürnberg, Universitätsklinikum Erlangen, Erlangen, Germany

[4] Bavarian Cancer Research Center (BZKF), Erlangen, Germany

[5] Comprehensive Cancer Center Erlangen-EMN (CCC ER-EMN), Erlangen, Germany

[6] Deutsches Zentrum für Immuntherapie (DZI), Erlangen, Germany

***Corresponding Author:** Prof. Dr. rer. nat. Susetta Finotto, Department of Molecular Pneumology, Friedrich-Alexander-Universität (FAU) Erlangen-Nürnberg, Hartmannstraße 14, Erlangen 91052, Germany.

Phone: +499131-8535883; E-mail: Susetta.Finotto@uk-erlangen.de

http://www.molekulare-pneumologie.uk-erlangen.de

Conflict of interest: The authors declare no conflict of interest on the matter described in this manuscript.

Supplementary Figures

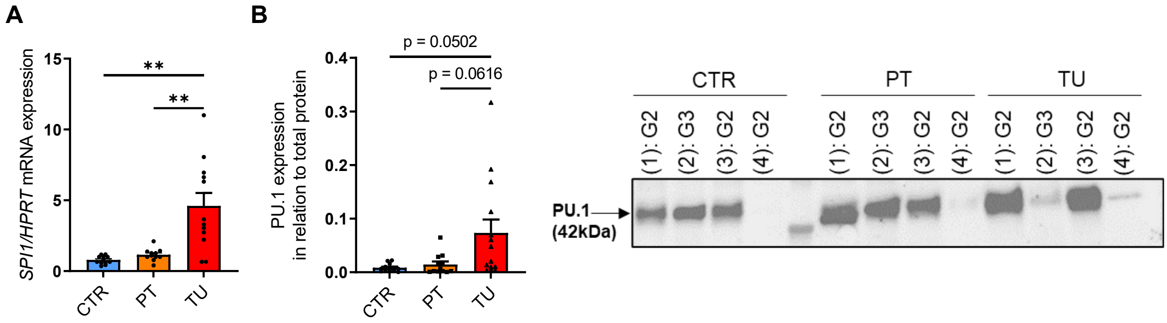

Supplementary Figure S1: **Increased PU.1 expression on mRNA and protein level in the tumoral region of the lung. A)** *SPI1* mRNA level from total lung cells was normalized on *HPRT* mRNA level (nCTR=12; nPT=11; nTU=12). **B)** Proteins from the different regions of the lung (CTR, PT, TU) were isolated directly from the tissue and analyzed for PU.1 protein expression by Western Blot. PU.1 protein levels were quantified relative to the total protein signal (using stain-free imaging) to ensure normalization across samples (nCTR=12; nPT=12; nTU=14). An exemplary western blot is shown on the right. N values are given per group. Bar charts indicate mean values +/- s.e.m. using Brown-Forsythe and Welch ANOVA test (A) or Kruskall-Wallis test (B). **p≤0.001.

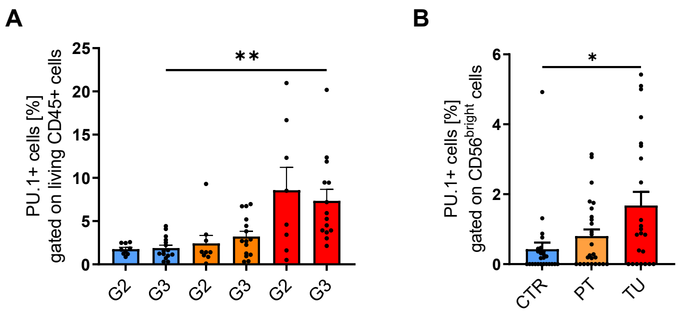

Supplementary Figure S2: **Analysis of PU.1 expression by flow cytometry. A)** Analysis of PU.1+ CD45+ cells (%) after classification by grading of tumor cell differentiation (nG2=8-9; nG3=14-15). **B)** Quantification of PU.1+ cells (%) gated on CD56^{bright} cells (nCTR=26; nPT=26; nTU=23). N values are given per group. Bar charts indicate mean values +/- s.e.m. using Kruskall-Wallist test. *p≤0.05; **p≤0.01.

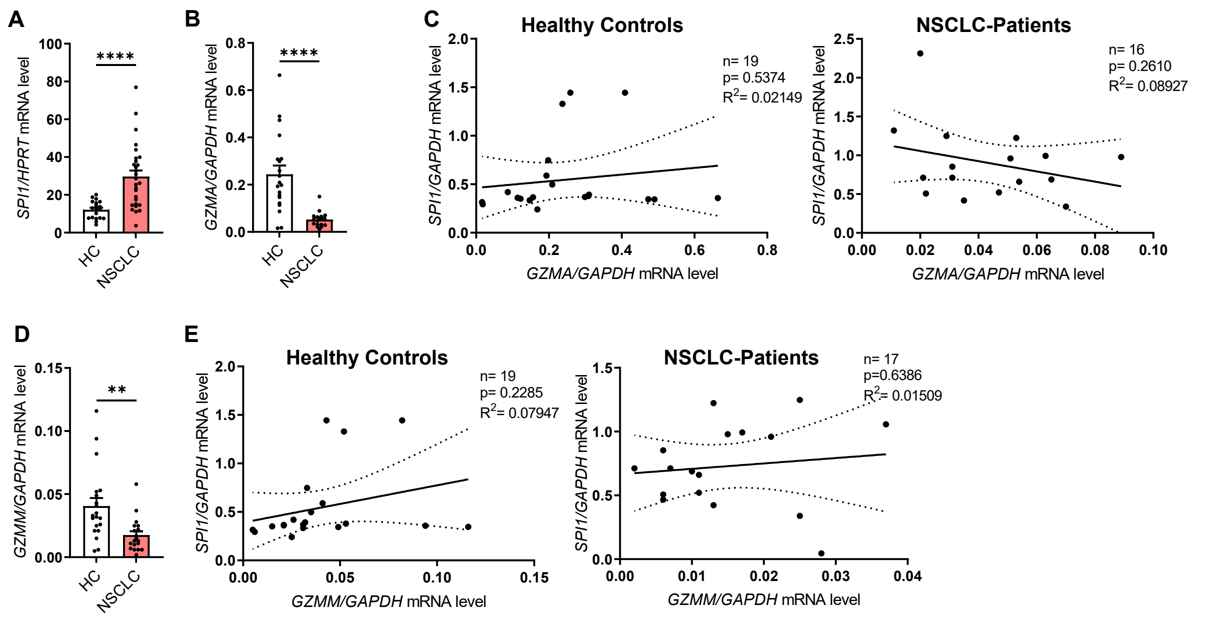

Supplementary Figure S3: **Analysis of PU.1 in PBMCs in context of effector functions. A)** *SPI1* mRNA level (nHC=20; nNSCLC=28) from PBMCs of healthy controls and NSCLC patients was normalized on *HPRT* mRNA level. **B)** *GZMA* mRNA level (nHC=20; nNSCLC=17) from PBMCs of healthy controls and NSCLC patients was normalized on *GAPDH* mRNA level. **C)** Correlation of *GZMA/GAPDH* mRNA levels with *SPI1/GAPDH* mRNA levels of healthy controls and NSCLC patients. **D)** *GZMM* mRNA level was normalized on *GAPDH* mRNA level (nHC=20; nNSCLC=19) and **E)** correlated with *SPI1*/*GAPDH* mRNA levels. N values are given per group. Bar charts indicate mean values +/- s.e.m. using Kruskall-Wallist test. **p≤0.01; ****p≤0.0001.

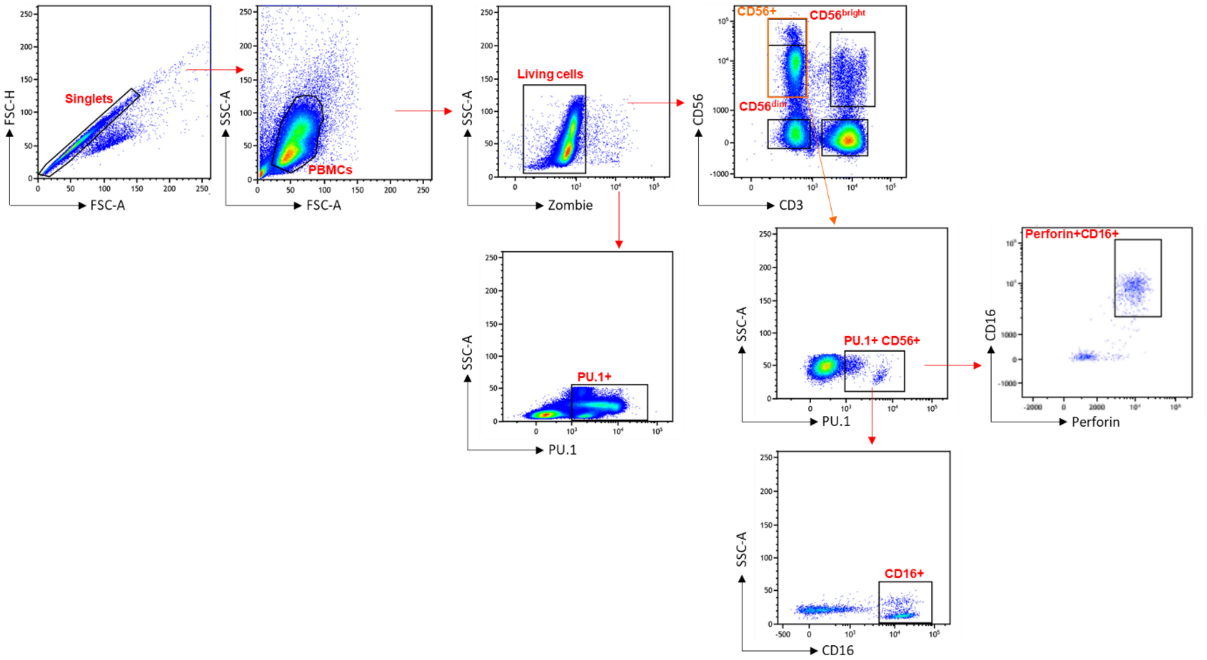

Supplementary Figure S4: Gating Strategy for human PBMCs. Peripheral blood mononuclear cells were isolated and analyzed by flow cytometry. First, doublets were excluded by gating single cells via FSC-A and FSC-H. From single cells, PBMCs were gated by FSC-A vs. SSC-A. Only living PBMCs were selected by gating on Zombie- cells. NK cells were identified as CD3-CD56+ cells with CD56^{dim} cells showing a lower signal for CD56 than CD56^{bright} cells. PU.1+ cells were gated on total living PBMCs as well as on CD56+ total cells and the CD56^{dim} and CD56^{bright} subpopulations. PU.1+ CD56+ total cells were additionally analyzed for CD16 expression and CD16+Perforin+ expression.

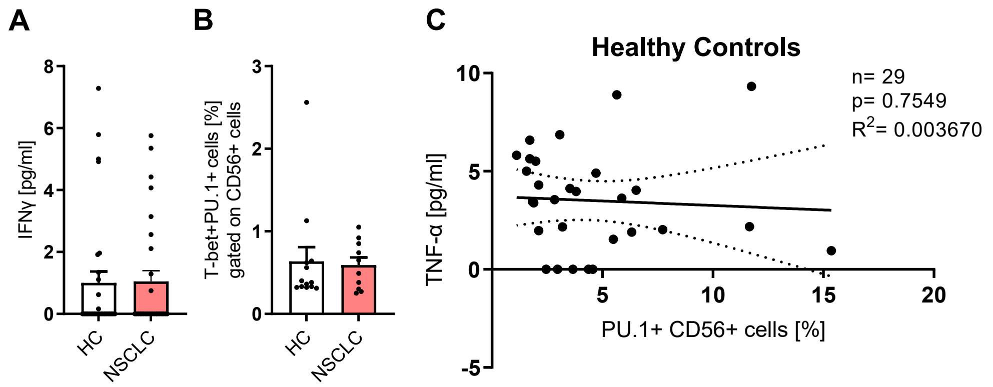

Supplementary Figure S5: PU.1 expressing NK cells are not connected to IFNγ secretion. A) IFN-γ levels [pg/ml] were measured in serum from healthy controls (n=30) and NSCLC patients (n=28). **B)** Quantification of PU.1+T-bet+ cells (%) gated on CD56+ PBMCs. **C)** TNF-α levels [pg/ml] in serum from healthy controls were correlated with PU.1+ CD56+ cells (%). Bar charts indicate mean values +/- s.e.m.

Supplementary Tables

Supplementary Table S1: Clinical data of NSCLC-cohort analyzed in this study

ID	Gender	Age	Histology	Grading	Tumor diameter [cm]	TNM	Smoking [PY]	Period of restriction [Years]
NSCLC 1	Male	79	ADC	G3	5	IIA	60	0
NSCLC 2	Male	63	ADC	G3	2,5	IA3	100	0
NSCLC 3	Female	70	ADC	G3	1,8 and 2,8	IIB	15	13
NSCLC 4	Male	60	ADC	G3	2,7	IA3	65	0
NSCLC 5	Female	64	ADC	G2	0,9	IA1	45	0
NSCLC 6	Male	61	ADC	G3	1,6	IA2	46	0
NSCLC 7	Female	77	ADC	G2	2,2	IA3	35	35
NSCLC 8	Female	55	ADC	G3	2,9	IIIA	30	0
NSCLC 9	Female	77	ADC	G3	7,2	IIIB	45	0
NSCLC 10	Female	74	ADC	G2	1,5 and 1,6	IIIA	0	0
NSCLC 11	Female	65	ADC	G3	3,3	IIIB	45	0
NSCLC 12	Male	64	ADC	G3	3,5	IB	100	0
NSCLC 13	Female	67	ADC	G3	1,9	IA2	200	0
NSCLC 14	Male	82	ADC	G2	2,7 (2x)	IIIA	0	0
NSCLC 15	Male	59	ADC-SCC	G3	10,5	IIIA	105	10
NSCLC 16	Female	69	ADC	G2	2	IA2	8	0,5
NSCLC 17	Female	67	ADC	G1	1,8	IA	40	28
NSCLC 18	Female	59	ADC	G3	0,8	IB	20	0
NSCLC 19	Male	81	SCC	G3	4,2	IIA	0	0
NSCLC 20	Male	69	ADC	G2	1,2	IA2	84	0
NSCLC 21	Male	64	ADC	G2	2,3	IA3	100	0
NSCLC 22	Male	61	ADC	G1	4,2	IIA	15	30
NSCLC 23	Female	49	SCC	G3	12	IIIA	30	0
NSCLC 24	Male	75	ADC	G2	1,3	IA2	1,6	0
NSCLC 25	Female	59	ADC	G2	2,7	IA3	0,5	11,5
NSCLC 26	Male	62	ADC	G3	2,5	IIIA	120	0
NSCLC 27	Female	67	SCC	G2	1,4	IV	40	11
NSCLC 28	Male	65	ADC	n.a.	n.a.	III	50	0
NSCLC 29	Male	77	ADC	G2	1,7	IA2	70	0
NSCLC 30	Female	79	SCC	G3	9	IIIA	1,5	0
NSCLC 31	Male	71	SCC	n.a.	2,7	IV	10	40
NSCLC 32	Male	82	ADC	G2	1,5	IA2	15	50
NSCLC 33	Male	56	ADC	G3	3,1	IB	40	0
NSCLC 34	Female	60	ADC	G2	1,7	IA2	45	0
NSCLC 35	Male	69	ADC-SCC	G3	2,1	IA3	1,25	0
NSCLC 36	Female	71	SCC	G3	4,2	IIA	35	18
NSCLC 37	Female	73	ADC	G2	3,3	IB	0	0
NSCLC 38	Male	71	SCC	G3	2,1	IA3	87	0
NSCLC 39	Female	80	SCC	G3	1,9	IA2	30-40	0
NSCLC 40	Female	83	ADC	G2	2,9	IB	0	0

NSCLC 41	Female	74	ADC-SCC	G3	1,5	IA2	84	0
NSCLC 42	Male	64	ADC	G2	2,1	IB	40-50	0
NSCLC 43	Male	73	SCC	G3	2,7	IA3	30	30
NSCLC 44	Female	67	ADC	G3	2,9	IA3	20	0
NSCLC 45	Male	67	ADC	G3	1,2	IA2	47	1
NSCLC 46	Male	83	SCC	G3	2,1	IA3	55	0
NSCLC 47	Male	65	SCC	G3	6,5	IIIB	25	0
NSCLC 48	Male	81	SCC	G3	8,5	IIB	17	46
NSCLC 49	Female	55	ADC	G1	1,2	IIIA	40	5
NSCLC 50	Male	67	ADC	G3	2,6	IIIA	35	0
NSCLC 51	Female	75	ADC	G2	2,3	IA3	40	0
NSCLC 52	Male	61	ADC	G2	2,5	IIIA	40-50	0
NSCLC 53	Female	67	ADC	G2	2,4	IA3	5	48
NSCLC 54	Female	71	ADC	G3	1,8	IA2	0	0
NSCLC 55	Female	73	ADC	G2	3,6	IB	3-4	5
NSCLC 56	Male	73	SCC	G3	7,1	IIIA	90	14
NSCLC 57	Male	61	ADC	G3	6,1	IIB	50	0
NSCLC 58	Female	77	ADC	G2	1,5	IIB	0	0
NSCLC 59	Female	83	ADC	G2	3,4	IB	0	0
NSCLC 60	Male	55	ADC	G3	2,4	IA3	30	0
NSCLC 61	Male	56	ADC	G2	2,6	IIIA	60	0

Abbreviations:

Histological classification: ADC= Lung Adenocarcinoma, SCC= Lung Squamous cell carcinoma;

Histopathological Grading: G1 = well differentiated, G2 = moderately differentiated, G3 = poorly differentiated;

n.a.=not applicable

TNM: T: describes the size of primary tumor, N: describes presence or absence and numbers of metastasis in the nearby lymph nodes, M: describes presence or absence of distant metastasis

PY: Pack Years; calculated by multiplying the number of packs of cigarettes (1 pack = 20 cigarettes) smoked per day by the number of years the person has smoked.

Supplementary Table S2: Clinical data of control subjects analyzed in this study

ID	Gender	Age	Smoking [PY]	Period of restriction [Years]
HC 1	Female	53	7	0
HC 2	Male	25	0	0
HC 3	Male	31	12	0
HC 4	Male	30	11,5	0
HC 5	Male	24	0	0
HC 6	Male	52	7	0
HC 7	Male	24	4	0
HC 8	Female	24	0	0
HC 9	Female	45	0	0
HC 10	Male	46	36	0
HC 11	Male	24	5	0
HC 12	Female	34	0	0
HC 13	Female	56	0	0
HC 14	Female	44	0	0
HC 15	Male	55	0	0
HC 16	Male	53	0,55	0
HC 17	Male	25	0	0
HC 18	Male	45	0	0
HC 19	Male	60	0	0
HC 20	Female	57	0	0
HC 21	Male	62	0	0
HC 22	Female	59	0	0
HC 23	Male	50	27,5	0
HC 24	Female	43	31,25	0
HC 25	Male	60	0	0
HC 26	Female	59	0	0
HC 27	Male	59	0	0
HC 28	Female	56	0	0
HC 29	Female	64	0	0
HC 30	Male	64	0	0
HC 31	Female	29	0	0
HC 32	Female	40	20	2,5
HC 33	Male	65	0	0
HC 34	Female	41	0	0

Abbreviations:

HC: Healthy controls
PY: Pack Years; calculated by multiplying the number of packs of cigarettes (1 pack = 20 cigarettes) smoked per day by the number of years the person has smoked.

Supplementary Table S3: FACS antibodies and dyes

Antigen	Fluorochrome	Clone	Company	Identifier
CD3	FITC	UCHT1	BioLegend	Cat# 300406
CD16	APC	eBioCB16	eBioscience	Cat# 17-0168-41
CD45	PerCP	2D1	BD Biosciences	Cat# 345809
CD56	PE/Cy7	5.1H11	BioLegend	Cat# 362509
CD279	APC-Fire750	EH12.2H7	BioLegend	Cat# 329953
T-bet	BV421	4B10	BioLegend	Cat# 644832
PU.1	PE	7C6B05	BioLegend	Cat# 658009
Perforin	BV421	deltaG9	BD Biosciences	Cat# 563393

Supplementary Table S4: List of primers used for Quantitative Real-Time PCR

Gene	Primer Sequence
h*GAPDH*	fw: 5´- AAA TCA AGT GGG GCG ATG CT - 3´<br>rev: 5´- CAA ATG AGC CCC AGC CTT CT - 3´
h*HPRT*	fw: 5´- TGA CAC TGG CAA AAC AAT GCA - 3´<br>rev: 5´- GGT CCT TTT CAC CAG CAA GCT - 3´
h*SPI1*	fw: 5´- GGC AAC CGC AAG AAG ATG AC - 3´<br>rev: 5´- CTT CGC CGC TGA ACT GGT AG - 3´
h*PRF1*	fw: 5´- GGT TCA CTG CCA CGG ATG - 3´<br>rev: 5´- ACA GGT GCC AAG GAG GTC - 3´
h*GZMA*	w: 5´- CCC TAT CCA TGC TAT GAC CC- 3´<br>rev: 5´- GCA CTA TTG TGA GTC CTG CC- 3´
h*GZMB*	fw: 5´- CGA CAG TAC CAT TGA GTT GTG CG - 3´<br>rev: 5´- TTC GTC CAT AGG AGA CAA TGC CC - 3´
h*GZMM*	w: 5´- CTC ACT GCA GAG AAA TGG CTC C -3´<br>rev: 5´- CCT TGA TGT GGA AGG TGA GAC C- 3´
h*TNF*	fw: 5´- CCC TGA AAA CAA CCC TCA GA - 3´<br>rev: 5´- AAG AGG CTG AGG AAC AAG CA - 3´
